# Nonemergent Percutaneous Coronary Intervention on an Unprotected Left Main Coronary Artery Supported with Impella® Heart Pump in Patients Ineligible for Surgical Revascularization

**DOI:** 10.1155/2019/9691753

**Published:** 2019-06-04

**Authors:** Perwaiz M. Meraj, Simon Dixon, Jeffery Moses, Karim Ibrahim, Andreas Schäfer, Ibrahim Akin, Jonathan Hill, Theodore Schreiber, William W. O'Neill

**Affiliations:** ^1^Northwell Health, Department of Cardiology, Manhasset, NY 11030, USA; ^2^Beaumont Health, Department of Cardiovascular Medicine, Royal Oak, MI 48073, USA; ^3^Columbia University Medical Center, New York, NY 10032, USA; ^4^Technische Universität Dresden, Department of Internal Medicine and Cardiology, Dresden, Germany; ^5^Hannover Medical Center, Department of Cardiology and Angiology, Hannover, Germany; ^6^Mannheim Medical School, Department of Cardiology and Angiology, Mannheim, Germany; ^7^King's College Hospital, London SE5 9RS, UK; ^8^Detroit Medical Center, Department of Cardiology, Detroit, MI 48201, USA; ^9^Henry Ford Medical Center, Department of Interventional Cardiology and Structural Heart, Detroit, MI, USA

## Abstract

**Objectives:**

We sought to assess if ineligibility to coronary artery bypass grafting (CABG) constitutes a risk factor in patients who underwent a nonemergent unprotected left main coronary artery (ULMCA) percutaneous coronary intervention (PCI) with prophylactic Impella® heart pump support.

**Background:**

ULMCA PCI in patients not deemed eligible for CABG is associated with significantly worse outcomes compared to ULMCA PCI in eligible patients.

**Methods:**

Patients from the cVAD Registry and the PROTECT II trial who underwent a nonemergent ULMCA PCI were identified. We compared in-hospital mortality and major adverse cardiac and cerebrovascular event (MACCE) rates as well as 30-day survival and MACCE rates between CABG ineligible and CABG eligible patients.

**Results:**

A total of 331 patients were included (293 Impella 2.5®, 38 Impella CP®); 227 were ineligible for CABG and 104 were eligible. Baseline characteristics were remarkable for a trend toward higher rate of chronic obstructive pulmonary disease in the ineligible patients. In-hospital mortality (3.52% vs. 5.77%; p=0.383) and MACCE (6.61% vs. 7.69%; p=0.816) rates as well as 30-day survival (92.0% vs. 93.4%; Log-Rank p-value =0.781) and MACCE (88.1% vs. 90.1%; Log-Rank p-value=0.648) rates were not different between the two groups.

**Conclusions:**

The results of our study suggest that prophylactic Impella support appears to mitigate the risks inherent to surgical ineligibility in patients undergoing a nonemergent ULMCA PCI. Our results require further investigation.

## 1. Introduction

The outcomes of percutaneous coronary intervention (PCI) and coronary artery bypass grafting (CABG) surgery for the treatment of left main coronary artery (LMCA) disease have been studied in recent randomized controlled trials [1, 2] and meta-analyses at the population-level data [3–5] as well as the individual patient-level data [6]. Although this coronary lesion is relatively infrequent at approximately 5% prevalence [7], LMCA PCI revascularization has experienced wider adoption and a significant improvement in outcomes [8].

Acute improvement in clinical outcomes has been demonstrated for PCI over CABG; however, the long-term results reveal clinical equipoise between the two strategies [1, 3]. Indeed, while repeat revascularization rates are still in favor of the CABG strategy, stroke rates are in favor of the PCI strategy. This holds true even in the subgroup of patients with LMCA disease as shown in the most recently published meta-analyses of randomized controlled trials [4, 5].

A PCI revascularization strategy is an acceptable alternative to CABG for patients with coronary disease of low and intermediate complexity, but the European and U.S. guidelines recommend CABG for those with high complexity coronary disease [9, 10]. Encouraging results from the EXCEL trial suggest that PCI is perhaps a preferred alternative to CABG in selected patients with LMCA disease who are candidates for either procedure [2]. Patients enrolled in this large-scale and well-conducted trial had a mean left ventricular ejection fraction (LVEF) above 56% at baseline, the overall use of hemodynamic support devices was less than 5%, and 100% were eligible to both revascularization strategies. Conversely, patients enrolled in the PROTECT II trial [11] had a mean LVEF below 24%, at least 64% were not surgical candidates, and per study protocol and according to enrolling investigators all the patients requiring hemodynamic support represent a sicker patient population. These higher risk patients, especially those ineligible for surgical revascularization, are not often studied and their outcomes are not well referenced or benchmarked. Indeed, several studies have shown that surgical ineligibility is associated with increased mortality in patients undergoing PCI on a diseased unprotected left main coronary artery (ULMCA) [12, 13]. More recently Sukul et al. demonstrated in a larger study no difference in outcomes between surgically ineligible patients and the rest of the patients who underwent PCI, except in the subgroup of patients who underwent PCI on ULMCA [14]. In these patients, surgical ineligibility was associated with significantly higher and unacceptable in-hospital mortality as high as 20%.

In this study, we sought to compare the outcomes of high-risk patients deemed ineligible for surgical revascularization and those eligible ones who underwent prophylactic support with Impella® heart pump for a nonemergent PCI on an ULMCA. The study results were presented as a poster in the Journal of American College of Cardiology in March 2018.

## 2. Methods

### 2.1. Patient Population

All consecutive patients enrolled in the cVAD Registry™ (catheter based ventricular assist device registry) who underwent prophylactic hemodynamic support with Impella® heart pump prior to a nonemergent PCI on an ULMCA were analyzed. In addition, similar patients enrolled in the PROTECT II trial [4] were included as well. The respective institutional review board of the sites participating in the cVAD registry reviewed the study protocol and authorized an informed consent waiver for the retrospective data extracted from the chart of patients included in this analysis. All the patients enrolled in the Protect II randomized controlled trial provided informed consent.

Investigators were asked to report in the case report form (CRF) of the aforementioned studies the recommendations of a surgical consultation when it occurred. The reason for ineligibility was not reported. In the PROTECT II trial investigators were asked to request a surgical consultation to prospectively assess patient eligibility for CABG. Although the CRF sections regarding surgical eligibility are identical in both the PROTECT II trial and the cVAD Registry™, in the latter the investigators and the staff chart abstractor at each site reviewed the patient charts including admission notes, consult notes, nursing notes, catheterization report, and discharge summary to identify and report the documentation of surgical ineligibility at any time before PCI. In our study, patients were considered ineligible for CABG when ineligibility was documented in the patient chart whether a surgical consultation occurred or not. All remaining patients were considered eligible for CABG including those who declined CABG following a surgical consultation recommending surgery. Patients who underwent an emergent PCI with or without cardiogenic shock and patients with history of CABG were excluded from this analysis.

### 2.2. Study Endpoints

The primary endpoint measured was all-cause in-hospital mortality rate. The secondary endpoint included in-hospital rate of major adverse cardiac and cerebrovascular events (MACCE) defined as death, stroke, myocardial infarction, and repeat revascularization with PCI or CABG. A Kaplan-Meier analysis was performed to estimate the 30-day MACCE and survival rates.

### 2.3. Device

The Impella® 2.5 and Impella® CP devices (Abiomed Inc., Danvers, Massachusetts, USA) are 12 French (Fr) and 14 Fr, respectively, microaxial pumps mounted on a 9 Fr catheter. They are inserted through the femoral artery using a modified Seldinger technique. The pump is advanced retrogradely across the aortic valve into the left ventricle under fluoroscopy guidance. Impella® 2.5 and Impella® CP devices can generate up to 2.5 L/min and 4.0 L/min of forward flow, respectively, directly in the ascending aorta. Heparinized dextrose fluid is purged through the pump and released in the general circulation at a rate of 4 – 12 mL/hr to prevent clot formation in the motor and early pump wear. The manufacturer recommends an activated thrombin time (ACT) of 160–180 seconds during pump support.

### 2.4. Statistical Analysis

Data are expressed as mean ± standard deviation (SD) or median with quartiles or frequencies as appropriate. A two-tailed unpaired t-test was used for parametric two-group comparisons on continuous variables. Alternatively, a Mann-Whitney test was used if the assumption of the normality of the distribution for the continuous variable could not be established. Pearson's *χ*^2^ test or Fisher exact test was used as appropriate for nominal data. In-hospital mortality is reported as the proportion of patients that were expired during the index hospitalization. A Kaplan-Meier estimate with a log-rank test was used to compare MACCE and survival rates up to 30 days between the ineligible patient group and the eligible patient group. Surviving patients were censored at 30 days or last known follow-up, whichever is earlier in this analysis. In order to identify potential confounding factors, a Cox proportional hazard regression for 30-day mortality and 30-day major adverse cardiac and cerebrovascular events (MACCE) was conducted. The model included covariates with a p value of 0.1 or less identified in the baseline characteristic comparative analysis between eligible and ineligible groups. All p-values were two-tailed and considered significant when the probability was less than 0.05. The statistical analyses for this report were performed using a JMP 10 software package (SAS Institute Inc., Cary, NC).

## 3. Results

A total of 331 patients, 297 consecutive patients from the cVAD Registry™ (supported between July 2008 and May 2015) and 34 from the PROTECT II trial (supported between January 2008 and October 2010), who underwent prophylactic hemodynamic support with an Impella® heart pump (293 Impella 2.5®, 38 Impella CP®) for a nonemergent PCI on an ULMCA were included. Of the 331 patients, 227 were ineligible for surgical revascularization and 104 were eligible. The baseline and procedural characteristics of the two groups were not different with the exception of a significantly higher rate of history of AICD/pacer implanted in the eligible patients and a trend toward higher rate of chronic obstructive pulmonary disease (COPD) in the ineligible patients. The baseline characteristics are presented in Table 1 and the procedural characteristics in Table 2.

**Figure 1:**
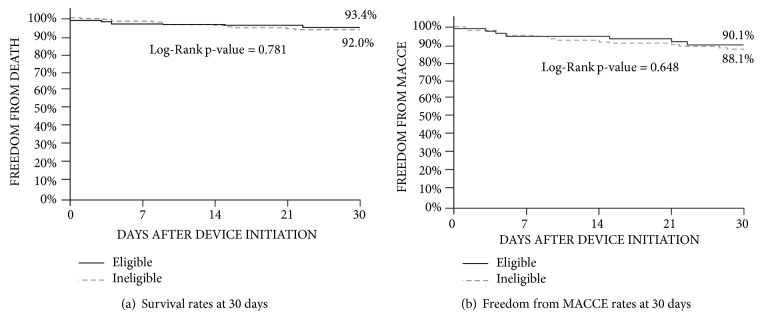
Kaplan-Meier analysis of estimated 30-day survival rate and freedom from MACCE rate. MACCE: major adverse cardiac and cerebrovascular events (death, stroke, myocardial infarction, and repeat revascularization).

The in-hospital mortality was not significantly different between the ineligible group and the eligible group (3.52% vs. 5.77%; p = 0.383). Furthermore, there was no difference between the two groups with regard to in-hospital MACCE (6.61% vs. 7.69%; p= 0.816) (Table 3), 30-day MACCE rate (88.1% vs. 90.1%; Long-Rank p-value = 0.648), or 30-day survival rate (92.0% vs. 93.4%; Long-Rank p-value = 0.781) (Figure 1). There were no differences in other in-hospital outcomes reported by the sites (Table 3).

## 4. Discussion

The present study of high-risk patients who underwent a nonemergent PCI on an ULMCA demonstrates that surgical ineligibility is not associated with worse in-hospital outcomes or worse 30-day survival when prophylactic support with Impella heart pump is undertaken.

Temporal trends of revascularization strategies in ULMCA stenosis reported recently by Park et al. show that more patients with ULMCA disease receive PCI and the gap in the treatment effect between PCI and CABG has decreased despite worsening of patient comorbidities and ULMCA stenosis complexity over time [8]. Furthermore, the advent of minimally invasive, potent, and rapidly deployable percutaneous left ventricular assist devices, such as the Impella heart pumps, has enabled interventional cardiologists to safely and effectively treat patients presenting with high-risk features for periprocedural complications [11].

Indeed, Schreiber et al. [15] reported recently a single-center experience which demonstrated favorable in-hospital and 30-day survival (98.43% and 97.74%, respectively) among patients with depressed LV function (28.74±15.55%) and multiple comorbidities (Society of Thoracic Surgeon (STS) morbidity score of 23.6±12.04) who underwent prophylactic Impella support for a ULMCA PCI. The predicted surgical mortality estimated by the STS score in these patients was lower than the STS mortality score of our patients (3.59±3.63 vs. 6.06±6.57), suggesting a less sick patient population who might have been eligible for surgical revascularization.

Prior studies have demonstrated that complex patients with ULMCA disease or multivessel coronary artery disease (CAD) who are ineligible for surgical revascularization have worse outcomes following a nonemergent PCI than those eligible for surgical revascularization [1, 2]. However, it is worth noting that these studies reported either a paucity of or no patients supported with percutaneous ventricular assist devices (pVAD). A recent study, conducted by Sukul et al. and including 99,370 patients who underwent a nonemergent PCI (<2% pVAD use) between June 2010 and December 2014 at 33 hospitals with on-site cardiac surgery, showed that PCI in surgical ineligible patients was generally safe except among patients who underwent ULMCA PCI. In this ULMCA PCI subgroup, surgically ineligible patients experienced significantly higher in-hospital mortality compared to non-ineligible patients (20.0% vs. 5.3%, p=0.02) and significantly higher incidence of cardiogenic shock during or following the index nonemergent PCI (25.0% vs. 5.1%, p=0.004) [3]. In contrast, when comparing outcomes between surgically ineligible patients in our study and those included in the Sukul et al., we find that our patients had significantly lower in-hospital mortality rate (3.5% vs. 20.0%, p=0.001). Furthermore, mortality rate in our entire cohort was comparable to that of the non-ineligible patients who underwent ULMCA PCI in the Sukul et al. report (6.9% vs. 5.3%, p=0.73). These comparisons suggest that prophylactic Impella support might be associated with improved in-hospital survival in patients ineligible for surgical revascularization probably by reducing the occurrence of cardiogenic shock during or following an ULMCA PCI.

In our study the baseline and procedural characteristics of the surgically ineligible and the surgically eligible patients were not different. A relevant exception was a trend (p=0.068) toward higher rate of COPD in the surgically ineligible patients. Conversely, the most recent landmark studies comparing the impact of surgical ineligibility in nonemergent ULMCA PCI showed that ineligible patients present with higher comorbid risk factors [12, 13].

Indeed, it has been shown that registries are usually not well designed to characterize surgically ineligible patients and often do not capture the reason for ineligibility [12–14]. McNulty et al. addressed the limitations of registries for adequate documentations of surgical candidacy and selection biases in nonemergent LMCA stenting. These investigators found that poor targets or conduits, cachexia or frailty, and malignancy were the most frequently reported reasons for ineligibility to CABG. However, these risk factors were not captured in our study. Other frequent reasons for ineligibility included advanced age, severe systolic dysfunction, and renal insufficiency. These were well balanced between the two groups in our study, probably because they represent the main risk factors that lead the physician to initiate prophylactic Impella support. Furthermore, in terms of risk assessment, it appears that features that connote surgical risk do not necessarily overlap with features defining PCI patients at high risk for periprocedural complications. Indeed, advanced age, severe lung disease, poor targets, severe systolic dysfunction, or renal insufficiency does not necessarily constitute a contraindication to PCI, especially if the patient undergoes protected PCI with prophylactic mechanical circulatory support [11].

## 5. Limitations

Several limitations regarding this study need to be considered when interpreting our results. First, the case report form of both the cVAD registry and the PROTECT II trial captured patient eligibility for surgical revascularization; however, the reasons for ineligibility were not captured. Second, although a surgical consultation was requested for a majority of patients included in this study, the remainder were considered by default as eligible for surgical revascularization in absence of additional patient information indicating ineligibility. Third, the low rate of events in conjunction with the sample size of our study does not confer an adequate statistical power to detect a difference between the two groups. Fourth, the retrospective and observational design of our study precludes us from drawing anything more than inferences and associations between the surgical status and the outcomes. Fifth, patient selection bias cannot be excluded due to differences in practice at enrolling sites and due to differences between patients enrolled in the cVAD and Protect II. Finally, a Cox proportional hazard regression conducted to identify potential confounding factors between the two groups did not identify any covariates associated with increased risk of death or MACCE at 30 days.

## 6. Conclusion

The results of our study suggest that prophylactic Impella support mitigates the risks inherent to surgical ineligibility in patients undergoing a nonemergent ULMCA PCI. Our results require further investigation, as more surgical ineligible patients need ULMCA PCI.

## Figures and Tables

**Table 1:** Baseline characteristics.

Characteristics	All	CABG ineligible	CABG eligible	p-value
	(N=331)	(N=227)	(N=104)	
*Demographics*				
Age	72.72±11.35 (331)	72.16±11.23 (227)	73.92±11.55 (104)	0.191
Gender - Male	67.07% (222/331)	69.60% (158/227)	61.54% (64/104)	0.166
BSA (m^2^)	1.94±0.27 (317)	1.96±0.28 (218)	1.90±0.26 (99)	0.058
*Baseline Characteristics*				
Hyperlipoproteinaemia	74.92% (245/327)	72.12% (163/226)	81.19% (82/101)	0.097
Hypertension	89.43% (296/331)	88.11% (200/227)	92.31% (96/104)	0.336
Diabetes Mellitus	48.16% (157/326)	47.35% (107/226)	50.00% (50/100)	0.719
CAD	75.00% (225/300)	75.36% (159/211)	74.16% (66/89)	0.884
Angina	35.43% (107/302)	34.76% (73/210)	36.96% (34/92)	0.794
Stroke	8.81% (26/295)	9.71% (20/206)	6.74% (6/89)	0.506
Renal Insufficiency	27.69% (90/325)	26.55% (60/226)	30.30% (30/99)	0.503
Dialysis	27.85% (22/79)	28.30% (15/53)	26.92% (7/26)	1.000
Liver Insufficiency	2.45% (7/286)	2.94% (6/204)	1.22% (1/82)	0.677
COPD	30.96% (100/323)	34.07% (77/226)	23.71% (23/97)	0.068
Arrhythmia	27.69% (90/325)	25.22% (57/226)	33.33% (33/99)	0.140
PVD	26.85% (87/324)	26.11% (59/226)	28.57% (28/98)	0.683
CHF	51.31% (157/306)	48.58% (103/212)	57.45% (54/94)	0.173
NYHA Class III/IV	62.28% (71/114)	64.00% (48/75)	58.97% (23/39)	0.685
Prior AICD/Pacer Implanted	13.94% (46/330)	10.18% (23/226)	22.12% (23/104)	0.006
Valvular Disease	17.05% (52/305)	16.98% (36/212)	17.20% (16/93)	1.000
Prior MI	47.99% (155/323)	44.89% (101/225)	55.10% (54/98)	0.115
Prior PCI	41.16% (135/328)	40.71% (92/226)	42.16% (43/102)	0.810
Prior CABG	0.00% (0/331)	0.00% (0/227)	0.00% (0/104)	- -
Surgical consultation was requested	58.23% (191/328)	66.08% (150/227)	40.59% (41/101)	<.001
LVEF	34.97±17.38 (293)	36.05±17.80 (199)	32.69±16.30 (94)	0.123
STS Mortality Score	6.09±6.57 (305)	6.15±6.83 (208)	5.96±6.01 (97)	0.813
STS Morbidity Score	30.25±17.56 (305)	30.26±18.23 (208)	30.21±16.12 (97)	0.980
*Hemodynamics*				
Heart Rate (bpm)	73.01±16.58 (324)	72.66±15.45 (221)	73.75±18.83 (103)	0.610
Systolic Blood Pressure (mmHg)	125.50±24.26 (323)	124.86±24.24 (219)	126.85±24.37 (104)	0.492
Diastolic Blood Pressure (mmHg)	68.37±15.20 (323)	68.00±15.45 (219)	69.13±14.69 (104)	0.532
Mean Arterial Pressure (mmHg)	88.12±16.42 (323)	88.24±16.53 (219)	87.87±16.27 (104)	0.849
*Hematology and Blood Chemistry*				
RBC (10^6^/*μ*L)	3.92±0.88 (297)	3.91±0.95 (201)	3.94±0.71 (96)	0.728
WBC (10^3^/*μ*L)	8.42±3.65 (300)	8.34±3.86 (204)	8.57±3.16 (96)	0.579
Hgb (g/dL)	12.47±10.33 (310)	12.82±12.38 (208)	11.77±3.41 (102)	0.260
HCT (%)	34.99±5.55 (308)	34.99±5.62 (207)	34.99±5.42 (101)	0.997
Platelet Count (10^3^/*μ*L)	205.03±66.81 (303)	204.05±68.84 (205)	207.10±62.66 (98)	0.711
Total Bilirubin (mg/dL)	0.79±0.97 (93)	0.75±0.94 (62)	0.89±1.05 (31)	0.500
Creatinine (mg/dL)	1.49±1.22 (268)	1.51±1.22 (183)	1.44±1.21 (85)	0.635
eGFR (mL/min/m^2^)	56.49±25.00 (128)	56.52±25.43 (86)	56.43±24.42 (42)	0.985
AST (U/L)	45.22±72.84 (101)	40.16±77.03 (70)	56.65±62.01 (31)	0.296
ALT (U/L)	33.96±47.52 (102)	28.90±18.27 (70)	45.03±80.19 (32)	0.269

AICD: automatic implantable cardioverter defibrillator; ALT: alanine aminotransferase; AST: aspartate aminotransferase; CABG: coronary artery bypass grafting; CAD: coronary artery disease; CHF: chronic heart failure; NYHA: New York Heart Association; COPD: chronic obstructive pulmonary disease; eGFR: estimated glomerular filtration rate; Hgb: hemoglobin; HCT: hematocrit; LVEF: left ventricular ejection fraction; MI: myocardial infarction; PCI: percutaneous coronary intervention; PVD: peripheral vascular disease; RBC: red blood cells; STS: Society of Thoracic Surgeons; TIMI: thrombolysis in myocardial infarction; WBC: white blood cells.

**Table 2:** Procedural characteristics.

Characteristics	All	CABG ineligible	CABG eligible	p-value
	(N=331)	(N=227)	(N=104)	
Number of diseased lesions per patient (>= 50% stenosis)	2.23±0.78 (331)	2.17±0.76 (227)	2.35±0.82 (104)	0.060
Lesion Location				
Left Main Coronary Artery	34.5% (353/1023)	35.1% (240/683)	33.2% (113/340)	0.577
Left Anterior Descending Coronary Artery	31.5% (322/1023)	31.6% (216/683)	31.2% (106/340)	0.943
Left Circumflex Coronary Artery	23.5% (240/1023)	22.4% (153/683)	25.6% (87/340)	0.273
Right Coronary Artery	10.6% (108/1023)	10.8% (74/683)	10.0% (34/340)	0.746
TIMI Flow Pre PCI				
0	1.3% (8/613)	1.6% (7/432)	0.6% (1/181)	0.447
1	1.6% (10/613)	2.1% (9/432)	0.6% (1/181)	0.295
2	12.7% (78/613)	13.2% (57/432)	11.6% (21/181)	0.690
3	84.3% (517/613)	83.1% (359/432)	87.3% (158/181)	0.223
Number of lesions treated	2.14±0.81 (331)	2.10±0.79 (227)	2.24±0.86 (104)	0.137
Number of stents placed	2.46±1.31 (328)	2.46±1.25 (225)	2.46±1.45 (103)	0.993
Patients with 1 vessel treated	0.3% (1/331)	0.0% (0/227)	1.0% (1/104)	0.314
Patients with 2 vessels treated	83.4% (276/331)	82.4% (187/227)	85.6% (89/104)	0.527
Patients with 3 vessels treated	16.3% (54/331)	17.6% (40/227)	13.5% (14/104)	0.423
TIMI Flow Post PCI				
0	0.5% (4/753)	0.8% (4/527)	0.0% (0/226)	0.322
1	0.5% (4/753)	0.4% (2/527)	0.9% (2/226)	0.588
2	0.8% (6/753)	0.9% (5/527)	0.4% (1/226)	0.674
3	98.1% (739/753)	97.9% (516/527)	98.7% (223/226)	0.571
Duration of device support (hour)	1.00 [0.13,72.75]	1.02 [0.13,50.37]	0.94 [0.33,72.75]	0.160

CABG: coronary artery bypass grafting; PCI: percutaneous coronary intervention; TIMI: thrombolysis in myocardial infarction.

**Table 3:** In-hospital outcomes.

Adverse Events	All	CABG ineligible	CABG eligible	p-value
	(N=331)	(N=227)	(N=104)	
MACCE	6.95% (23/331)	6.61% (15/227)	7.69% (8/104)	0.816
Death	4.23% (14/331)	3.52% (8/227)	5.77% (6/104)	0.382
Myocardial Infarction	2.42% (8/331)	2.64% (6/227)	1.92% (2/104)	1.000
Stroke	0.00% (0/331)	0.00% (0/227)	0.00% (0/104)	- -
Repeat Revascularization	0.60% (2/331)	0.44% (1/227)	0.96% (1/104)	0.530
Acute Renal Dysfunction	4.83% (16/331)	3.52% (8/227)	7.69% (8/104)	0.164
Aortic Valve Regurgitation	0.00% (0/331)	0.00% (0/227)	0.00% (0/104)	- -
Hypotension During Support	3.93% (13/331)	4.41% (10/227)	2.88% (3/104)	0.762
Cardiopulmonary Resuscitation or Ventricular Arrhythmia	2.72% (9/331)	2.20% (5/227)	3.85% (4/104)	0.470

CABG: coronary artery bypass grafting; MACCE: major adverse cardiac and cerebrovascular events (death, stroke, myocardial infarction, and repeat revascularization).

## Data Availability

The cVAD registry data used to support the findings of this study are available from the corresponding author upon request; however, like any other prospective registry, they are held by the cVAD executive committee.
